# *Mycobacterium tuberculosis* H37Rv LpqG Protein Peptides Can Inhibit Mycobacterial Entry through Specific Interactions

**DOI:** 10.3390/molecules23030526

**Published:** 2018-02-27

**Authors:** Christian David Sánchez-Barinas, Marisol Ocampo, Magnolia Vanegas, Jeimmy Johana Castañeda-Ramirez, Manuel Alfonso Patarroyo, Manuel Elkin Patarroyo

**Affiliations:** 1 Fundación Instituto de Inmunología de Colombia (FIDIC), Carrera 50 No. 26-20, Bogotá 111321, Colombia. christian5_16@hotmail.com (C.D.S.-B.); magnolia.vanegas@urosario.edu.co (M.V.); jeimmyjohana93@gmail.com (J.J.C.-R.); mapatarr.fidic@gmail.com (M.A.P.); mepatarr@gmail.com (M.E.P.); 2 Basic Sciences Department, School of Medicine and Health Sciences, Universidad del Rosario, Carrera 24 No. 63C-69, Bogotá 111321, Colombia; 3 Pathology Department, Faculty of Medicine, Universidad Nacional de Colombia, Carrera 45 No. 26-85, Bogotá 111321, Colombia

**Keywords:** LpqG, lipoprotein, *Mycobacterium tuberculosis*, vaccine, synthetic peptide, Rv3623, mycobacterial entry inhibition

## Abstract

*Mycobacterium tuberculosis* is the causative agent of tuberculosis, a disease causing major mortality worldwide. As part of a systematic methodology for studying *M. tuberculosis* surface proteins which might be involved in host-pathogen interactions, our group found that LpqG surface protein (Rv3623) found in *M. tuberculosis* complex strains was located on the mycobacterial envelope and that peptide 16661 (^21^SGCDSHNSGSLGADPRQVTVY^40^) had high specific binding to U937 monocyte-derived macrophages and inhibited mycobacterial entry to such cells in a concentration-dependent way. A region having high specific binding to A549 alveolar epithelial cells was found which had low mycobacterial entry inhibition. As suggested in previous studies, relevant sequences in the host-pathogen interaction do not induce an immune response and peptides characterised as HABPs are poorly recognised by sera from individuals regardless of whether they have been in contact with *M. tuberculosis*. Our approach to designing a synthetic, multi-epitope anti-tuberculosis vaccine has been based on identifying sequences involved in different proteins’ mycobacteria-target cell interaction and modifying their sequence to improve their immunogenic characteristics, meaning that peptide 16661 sequence should be considered in such design.

## 1. Introduction

Tuberculosis is an air-born infection caused by the acid-alcohol resistant *Mycobacterium tuberculosis* (*Mtb*) bacillus and other *Mycobacterium tuberculosis* complex (MTC) strains [1]. It has been estimated that some 2 to 3 thousand million people worldwide are infected by the bacterium in a dormant/latent state and 6.3 million tuberculosis-related deaths were reported in 2016 [2]. The only licensed vaccine’s (BCG—*Mycobacterium bovis* Bacillus Calmette Guérin) variable efficacy against tuberculosis, together with the appearance of drug resistance, has driven the search for new vaccines against this disease [3,4,5]. In spite of arduous research by groups worldwide, attempts at effective vaccine design have been unsuccessful because factors such as virulence, pathogenicity and resistance to antibiotics have hampered such work. Multiple drug-resistant strains’ incidence and HIV co-infection necessitate urgent control measures through the development of new drugs and vaccines [6].

*Mtb* is a facultative pathogen which can survive within macrophages, acting as host cells [7]; this is why some research has focused on blocking pathogen-host recognition because when the bacillus enters a target cell it can evade the immune response and replicate freely within the macrophages [8,9]. Defining mycobacterial cell envelope composition and organisation is a challenge because it is a complex structure where peptidoglycans, arabinogalactans and mycolic acids predominate, non-covalently surrounding an outer capsule of proteins and polysaccharides [10]; clarifying protein make-up would thus help in understanding important aspects of *Mtb* pathogenicity and facilitate directing new research regarding pathogen-host interaction study. Research has shown that the envelope consists of cell membrane, a cell wall and a layer similar to an external capsule. It has been proven that *Mtb* cytoplasmic membrane contains many proteins involved in the bacterium’s physiology, including integral proteins having transmembrane domains and peripheral proteins involved in metabolic processes and their pathogenicity [11,12,13]. Lipoproteins are a functionally diverse type of membrane protein; they are anchored to mycobacterial membrane; these proteins’ signal peptides promote their export and post-translational modification towards cell envelope. Lipoproteins are characterised by having a sequence known as a “lipobox” at their C-terminal extreme which anchors a protein to extracellular surface; they have been previously synthesised as pre-prolipoproteins and mature by post-translational change [14,15]. The LpqG lipoprotein (Rv3623) has been described as probably conserved, having an unknown function, classified in the cell wall and cell processes category [12]; it shares conserved domains with bacteria outside and within the MTC. The *lpqG* gene is an orthologue of *M. bovis*, *M. leprae*, *M. marinum* and *M. smegmatis* strains.

Amongst *Mycobacterium tuberculosis* H37Rv virulent strain membrane proteins obtained by Triton X-114 phase separation, LpqG is on the list of the 10 most frequently observed lipoproteins on external membrane, although currently lacking a defined function. This highlights the lack of knowledge regarding the mycobacterium’s possible fundamental roles for a major lipoprotein. This lipoprotein has only been identified in *M. tuberculosis* fractions (i.e., not in *M. bovis* or *M. bovis* BCG Pasteur) and it has been found that the homologous gene in *M. bovis* lacks the signal peptide and that the lipobox motif has 207 bp less at the N-terminal extreme, suggesting no export towards protein membrane in these mycobacteria [16,17].

The present study regarding the LpqG protein describes our approach to the search for antigens when designing a synthetic anti-TB vaccine, involving peptide sequences of *Mtb* surface proteins having high specific infection target cell binding capacity, inhibiting *mycobacterium* entry in vitro and having certain structural elements [18,19,20,21]. Monocyte cell binding region have been identified at the protein’s N-terminal extreme and in epithelial cells’ central region; one such HABP has inhibited *Mtb* entry in a concentration-dependent manner.

## 2. Results

### 2.1. Bioinformatics Analysis

BLAST local alignment of sequences led to identifying a conserved protein domain SIMPL (signalling molecule that associates with the mouse pelle-like kinase) for LpqG, which has a tumour necrosis factor-specific regulator of nuclear factor-kappaB activity. It is conserved in different MTC strains and species having 100–98% sequence identity with *Mtb* H37Rv, *Mtb* H37Ra, *M. bovis* and *M. canettii* strains and *Mtb* T46, CDC551, T17 and C clinical isolates [22].

ProtParam (Table 1) predicted physicochemical properties, giving molecular weight, stability index (SI), isoelectric point, suggest that it is a potent immunogen; LpqG Gravy index score (average hydrophobicity and hydrophilicity) indicated low membrane association, characteristic of hydrophilic proteins [23,24]. Regarding its subcellular location, although tools have been validated for use regarding *Mtb* proteins, results differ according to the tool being used; however, the lipobox motif confirms Gpos-mPLoc prediction that LpqG should be considered a membrane protein [25]. SignalP 4.0 software predicted that LpqG would be exported by classical secretion, would have a signal peptide (0.2 C-score) and a cleavage site between Ser^25^ and His^26^ amino acids (aa); however, LipoP v 1.0 predicted a signal peptide cleavage site between Gly^22^ and Cys^23^, having a significantly high score (25.17). It was also determined that LpqG would have no transmembrane regions; topological prediction gave an extracellular region from aa 26, following the signal peptide [26].

### 2.2. lpqG Gene Presence and Transcription

**Figure 1:**
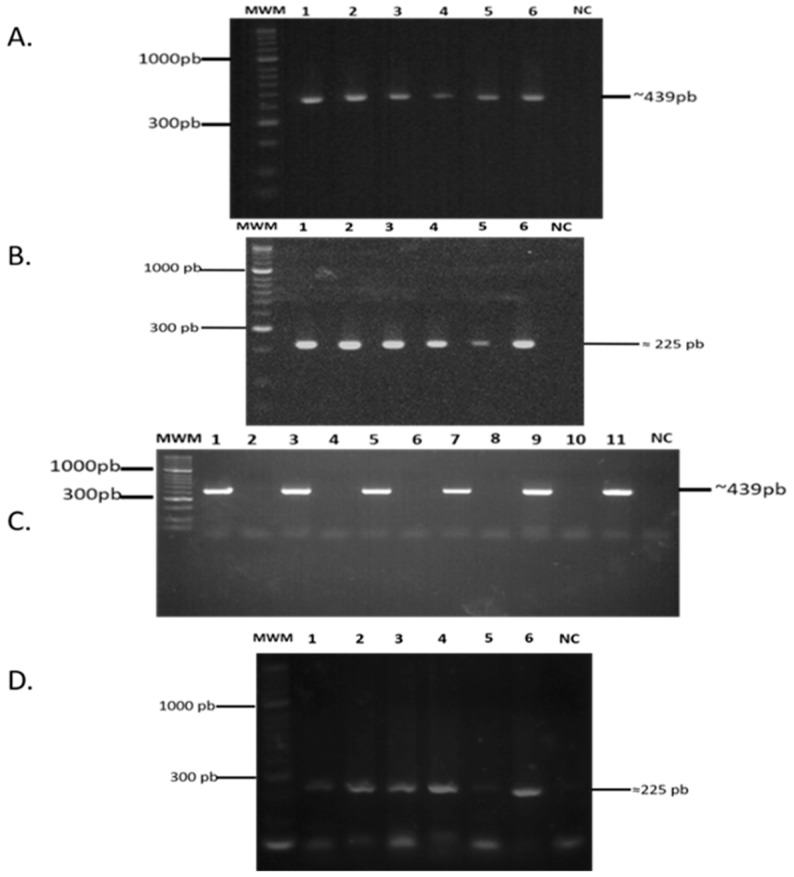
*rv3623/lpqG* gene presence and transcription. (**A**) Amplification of *hsp65* gene from gDNA isolated from: 1. *Mtb* H37Rv; 2. *Mtb* H37Ra; 3. *M. bovis*; 4. *M. bovis* BCG; 5. *M. smegmatis*; 6. Positive PCR control; *NC: Negative PCR control.* MWM: 50 bp molecular weight marker; (**B**) Amplification of *rv3623* gene from gDNA isolated from: 1. *Mtb* H37Rv; 2. *Mtb* H37Ra; 3. *M. bovis*; 4. *M. bovis* BCG; 5. *M. smegmatis*; 6. Positive PCR control; NC: Negative PCR control; (**C**) *hsp65* gene amplification from cDNA of: 1. *Mtb* H37Rvplus synthesis; 2. *Mtb* H37Rvminus synthesis; 3. *Mtb* H37Raplus synthesis; 4. *Mtb* H37Raminus synthesis; 5. *M. bovis* plus synthesis; 6. *M. bovis* minus synthesis; 7. *M. bovis* BCGplus synthesis; 8. *M. bovis* BCGminus synthesis; 9. *M. smegmatis* plus synthesis; 10. *M. smegmatis* minus synthesis; 11. Positive PCR control (*Mtb* H37RvgDNA); NC: Negative PCR control; MWM: 50 bp molecular weight marker; (**D**) *rv3623* gene amplification from cDNA of: 1. *Mtb* H37Rv; 2. *Mtb* H37Ra; 3. *M. bovis*; 4. *M. bovis* BCG; 5. *M. smegmatis*; 6. Positive PCR control; NC: Negative PCR control. MWM: molecular weight marker.

The gDNA samples had integrity, as shown by 439 bp *hsp65* gene presence (Figure 1A). Figure 1B shows a 325 bp amplification product, evidencing *lpqG* gene presence in *Mtb* H37Rv, *Mtb* H37Ra, *M. bovis* and *M. bovis* BCG (MTC strains) and *M. smegmatis* (a non-pathogenous strain). The hsp65 constitutive gene was also used as transcription control for each mycobacterial strain, confirming that there had been no type of gDNA-related contamination (Figure 1C). A 225 bp amplification fragment was observed in *M. tuberculosis* H37Ra; *M. bovis* and *M. bovis* BCG strains, and with less intensity in *M. tuberculosis* H37Rv and *M. smegmatis* strains, determining that the *lpqG* gene was transcribed in normal mycobacterial culture conditions during logarithmic growth phase (Figure 1D).

### 2.3. LpqG Protein Was Present on Mtb Surface

**Figure 2:**
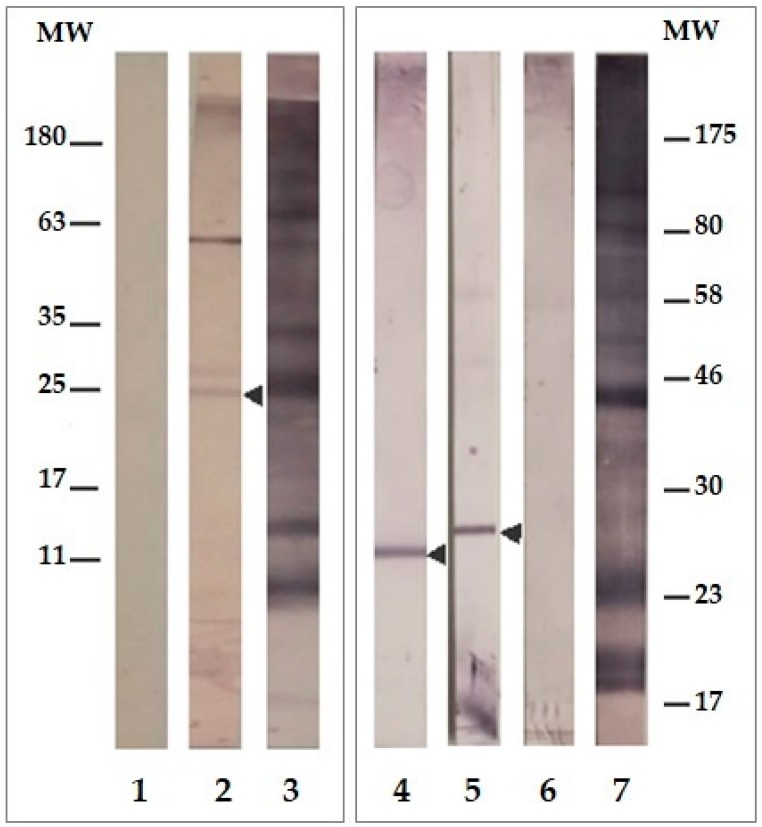
Western blot recognition of Rv3623 in *M. tuberculosis*. Lane 1: pre-immune sera. Lane 2: post-third immunisation of the 16660/16665/BSA mixture against lysed *Mtb* H37Rv. Lanes 3 and 7: recognition of lysed *Mtb* H37Rv by hyper-immune sera control (obtained by inoculating mice with *Mtb* H37Rv sonicate). Identification of membrane (lane 4), wall (lane 5) and cytosol proteins (lane 6) lysed from H37Rv by sera from mice immunized with the 16660/16665/BSA mixture (25 µg antigen was added to each lane).

Polyclonal sera showed that LpqG protein presence in *Mtb* H37Rv lysate (Figure 2) was recognised as a 25 kDa band having the protein’s theoretical weight (24.8 kDa). The protein was found to be present in mycobacterial wall and membrane fractions but not in its cytosol. No pre-immune sera recognition was observed.

### 2.4. Synthetic Peptides Bound Specifically to A549 and U937 Cells

**Figure 3:**
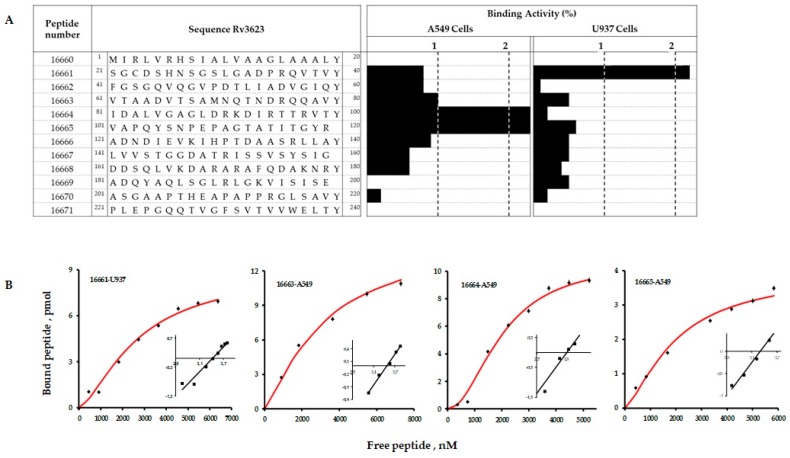
(**A**) synthetic peptides having high specific binding to A549 and U937 cells. Tyrosine (Y) was added to the N-terminal extreme of some of them to enable NaI^125^ radio-labelling. The black bars show specific target cell binding percentage; a peptide having greater than or equal to 1% binding was considered a HABP; (**B**) saturation curves for peptide 16661 in the U937 line and peptides 16663, 16664 and 16665 for A549 cells. Analysing Hill coefficients facilitated obtaining affinity constants and the maximum amount of sites per cell from the curves. Inset: the abscissa is Log F on the Hill Plot, and the ordinate is Log [B/Bmax − B], Bmax being the maximum amount of bound peptide, B the bound peptide and F free peptide.

Receptor-ligand assays led to identifying high specific target cell binding sequences in the LpqG protein, used as shown in Figure 3A. Three HABPs were identified for the A549 cell line in the region between peptides 16663 and 16665 (^61^VTAADVTSAMNQTNDRQQAVYIDALVGAGLDRKD IRTTRVTYVAPQYSNPEPAGTATITGYR^120^) and one HABP named 16661 for the U937 line (^21^SGCDS HNSGSLGADPRQVTVY^40^).

Saturation curves were used for determining HABP-cell line interaction physico-chemical constants (Figure 3B). A549-HABPs interaction dissociation constants (K_D_) were between 2000 nM–2800 nM and 3 × 10^6^–9 × 10^6^ receptors per cell, whilefor the HABP-U937 cell line interaction was found K_D_ 2800 nM and 6 × 10^6^ receptors per cell; the nature and the identity of possible cell receptors has yet to be established. Hill coefficients (n_H_) for these interactions were greater than one, this being characteristic of positive cooperativity interactions. Table 2 lists HABP-cell interaction constant values.

### 2.5. Inhibiting Mycobacterium tuberculosis Entry to Target Cells

**Figure 4:**
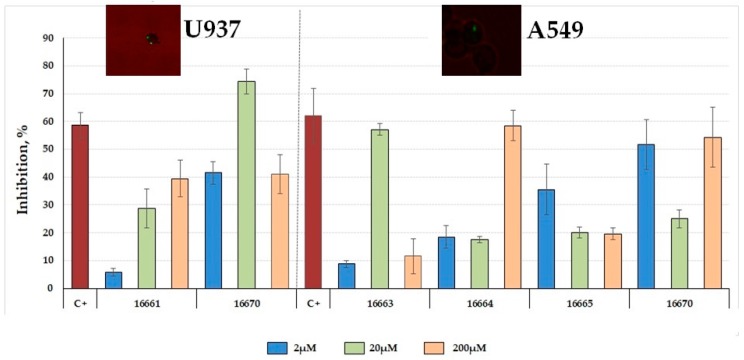
Inhibiting entry to A549 cells and U937 cells. Inhibition assay regarding *Mtb* invasion of A549 and U937 cells; 2, 20 and 200 µM concentrations were used for both cell lines. *Mtb*H37Rv lysate at 200 µg/mL concentration was used as inhibition control (C+). Mycobacteria within A549 and U937 cells can be observed in the fluorescence microscopic image (100×).

Only 16661, 16663, 16664 and 16665 peptide (identified as HABPs) capability to block mycobacterial entry to each cell line was determined (i.e., having specific binding). *Mtb* H37Rv lysate control had around 60% inhibition for both cell lines (Figure 4). HABP 16661 inhibited mycobacterial entry to monocyte-derived U937 macrophages in a concentration-dependent manner. Peptide 16663 inhibited mycobacterial entry to A549 cells regardless of concentration (maximum 57% at 20 µM concentration) whilst HABPs 16664 and 16665 had the same pattern (16664 inhibiting entry by around 59% at 200 µM and 16665 by 36% at 2 µM). Peptide 16670 (non-HABP) inhibited mycobacterial entry to both cell lines, regardless of concentration. LpqG peptides had no cytotoxic effect on the target cells used, except for peptides 16661 (4% cytotoxicity) and 16670 (6%) on A549 cells (data not shown).

### 2.6. LpqG Peptide Antigenicity

**Figure 5:**
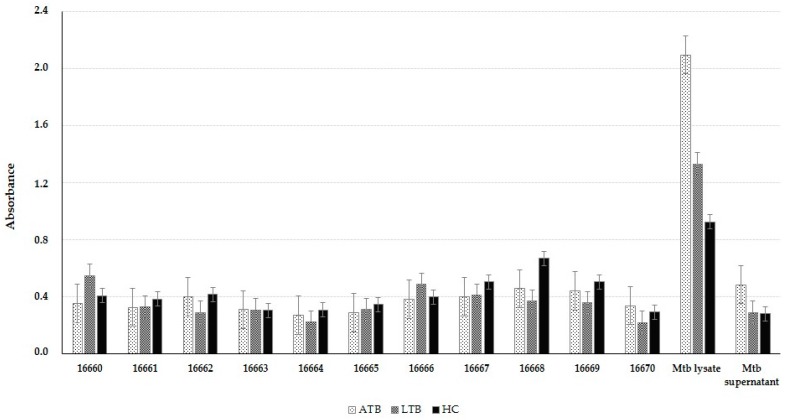
Antigen recognition of Rv3623’s peptides evaluated in sera from patients having active tuberculosis (ATB), latent tuberculosis (LTB) and healthy patients (HC). PBS was used as negative control and *Mtb* H37Rv lysate and Sauton medium supernatant proteins released as antigen were also used. Positive control involved using mouse polyclonal sera against peptides 16665 and 16660.

Figure 5 shows that even though response was highly dispersed, ATB and LTB patients’ sera contained more antibodies recognising *Mycobacterium tuberculosis* H37Rv lysate than that from healthy individuals (HC). ATB patients had more antibodies against mycobacterial lysate than LTB patients; there was a significance difference between ATB patients and HC individuals’ recognition. HC and LTB patients’ serum had similar recognition of *Mtb* H37Rv culture supernatant proteins, though without significant differences; there was greater recognition in ATB patient’s serum.

Dunnett’s multiple comparison test gave no statistically significant differences regarding serarecognition of each peptide, even though absorbancewas above the 0.086 threshold (calculated in the absence of antigen). LpqG protein peptide recognition was as low as that shown for mycobacterial supernatant for all sera used. HABP recognition was less than that obtained for the other protein peptides. Positive control using mouse polyclonal sera against peptides 16665 and 16660 confirmed thatpeptide binding to the plate was efficient.

### 2.7. LpqG Protein Peptides’ Secondary Structure

**Figure 6:**
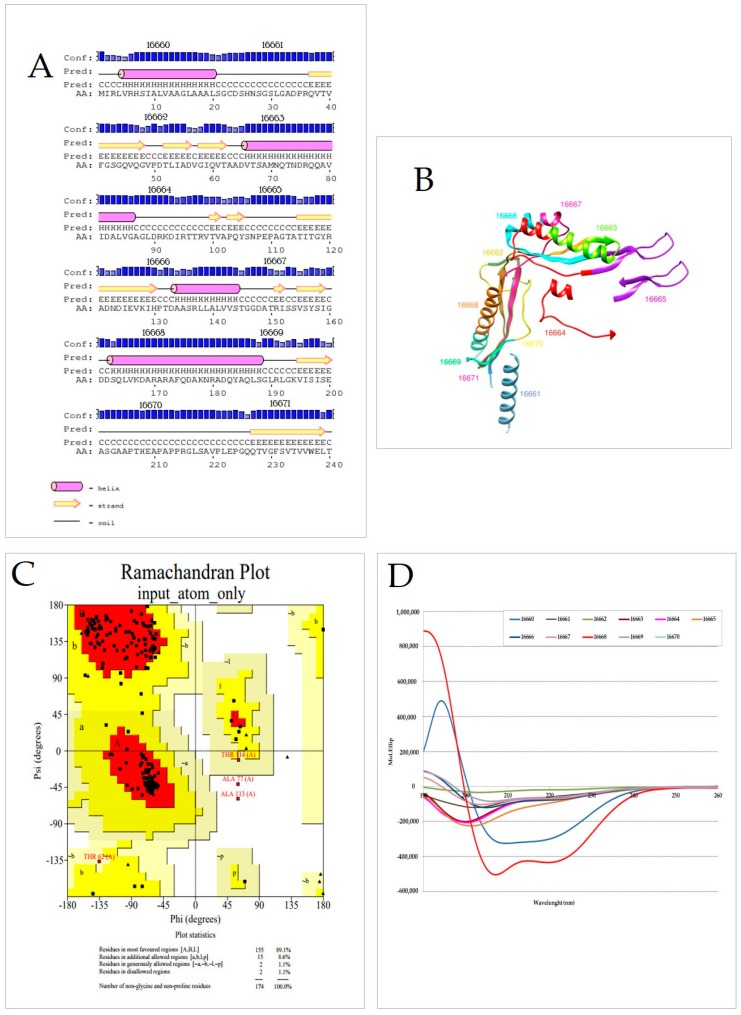
Bioinformatics prediction **of** Rv3623 structure (**A**) secondary structure determined by the PSI-PRED server regarding amino acid structure; (**B**) tertiary structure model of Rv3623 structure from synthetic peptides in *Mtb* Rv3623 protein 3D structure modelled in Pyre^2^ and validated in Swiss model and AMBER; Chimera 1.11.2 was used for constructing this this model; (**C**) Ramachandran Plot for evaluating the structural model; red areas indicate core regions and yellow allowed regions; (**D**) Rv3623 protein secondary structure analysis by CD molar ellipticity.

PSIPRED server’s predicted LpqG protein secondary structure as having three α-helix regions (containing peptides 16660, 16663 and 16668) and two β-sheet-rich regions (16662 and 16666) (Figure 6A). A 0.54 QMEAN score was obtained when protein 3D structure was modelled in Phyre^2^ [27] and validated in Swiss model and AMBER (Figure 6B) [28,29] (validated and processed just from peptide 16662). When the model was evaluated using Ramachandran Plot it gave 89.1% for the most favoured regions, thereby obtaining reliable structure with the test (Figure 6C). Residue aa location gave 8.6% in additional allowed regions and 1.1% generously allowed regions, giving an energetically stable structure.

Peptide 16661 had to be designed by Chimera 1.11.2 (based on CD experimental results) to provide a model covering most of the protein. AMBER was used for energy minimisation to improve and obtain better model concordance. The A549 cell binding region was exposed on the predicted model’s exterior.

The experimental approach for defining LpqG protein peptide secondary structure involved using CD [30,31]. HABPs 16661 (^21^SGCDSHNSGSLGADPRQVTVY^40^), 16663 (^61^VTAADVTSAMNQ TNDRQQAVY^80^) and 16664 (^81^IDALVGAGLDRKDIRTTRVTY^100^) had α-helix structure elements, characterised by having a 196 nm maxima and two minima (208 nm and 220 nm), whilst HABP 16665 (^101^VAPQYSNPEPAGTATITGYR^120^) had no defined structural elements (Figure 6D). The structural elements could not be associated with peptide sequence binding and inhibition functions. The results shown here only include unmodified peptide sequences (i.e., without glycosylation or acylation).

Regarding each peptide’s experimentally determined structural elements, great similarity was found with the protein’s predicted three-dimensional structure (Figure 6B) although there were appreciable differences concerning secondary structure prediction (Figure 6D).

## 3. Discussion

Lipoproteins forming part of the mycobacterial cell envelope play an important role in pathogen pathogenicity and its active biology and it has been suggested that they are involved in host-pathogen interaction. Characterising LpqG protein (Rv3623) in the present study contributes towards the search for *Mtb* protein sequences which could be involved in such interaction and which could be included when designing a synthetic anti-TB vaccine. In spite of not having an established function, it has been shown that LpqG is present in mycobacterial membrane and wall fractions, thereby making it of special interest from the functional point of view regarding interaction with infection target cells.

Following our methodological approach, *lpqG* gene presence and transcription (tested in normal culture conditions for strains inside and outside the MTC) suggested that it is not related to virulence but may be involved in mycobacterial physiological activity, thereby agreeing with that found in computational analysis predicting the gene’s presence in clinical isolates and pathogenic and non-pathogenic strains.

The LpqG protein has a signal peptide (^1^MIRLVRHSIALVAAGLAAALSGCD^24^) containing a conserved lipobox motif in all MTC strains (except for *M. bovis* BCG). The lipobox motif consists of a combination of four aa (LVI/ASTVI/GAS/C), functioning as a recognition site for modification with lipids in conserved cysteine residues and being essential for protein export to the membrane by classical secretion route [32], thereby facilitating its role in interaction with infection target cells. Modifying lipoprotein precursor proteins (i.e., LpqG) is mediated by the action of enzymes such as diacylglycerol-phosphatidylglycerol-pre-prolypoproteintransferase (Lgt), adding a diacylglycerol to the thiol group’s conserved cysteine. The Lsp A enzyme (prolipoprotein signal peptidase or signal peptidase II) then acts by splitting the signal peptide upstream of the cysteine; the phospholipid-apolipoprotein N-acyltransferase (Lnt) described for Gram-negative bacteria immediately adds the third acyl residue to the amino group to modify the cysteine and thus become transported through the periplasm. It has been suggested that there is a complex lipoprotein transport system for mycobacteria via the mycolic acid layer [14]. LpqG protein location on *Mtb* H37Rv envelop in normal culture conditions could be associated with its role in host-pathogen interaction.

LpqG protein location on the mycobacterial membrane and wall suggest its possible role in infection target cell interaction; bands having greater molecular weight recognised with less intensity could be attributed to the glycosylations predicted by the NetOGlyc 3.0 server [33].

The region between aa Val^61^ and Arg ^120^ in the LpqG sequence was identified as a high specific alveolar epithelial cell line (A549) binding region and HABP 16661 sequence (^21^SGCDSHNSGS LGADPRQVTVY^40^) for macrophages (U937); these peptides could be able to occupy the binding sites of cells necessary for *Mtb* entry. It should be stressed that additional assays are needed for characterising the target cell surface receptors to which the peptides bound. Only HABP 16661 inhibited mycobacterial entry to macrophages (U937) in vitro in a concentration-dependent manner, by up to 40%, without having a cytotoxic effect on cell interaction. On the other hand, peptide 16670 was not of interest, despite inhibiting *Mtb* entry, as it did not have high specific target cell binding capability, its action was due to unspecific effects, and it could be of interest in developing molecules having a therapeutic but not prophylactic nature. According to the proposed methodology, only HABPs inhibiting mycobacteria entry to target cells would be of interest when designing of a synthetic anti-tuberculosis vaccine.

Bioinformatics tools predicted protein secondary and tertiary structure, HABPs being exposed on such conformation; peptide 16661 was not located as part of such prediction even though it was confirmed that most structural elements found experimentally in protein peptides coincided with the theoretical prediction of the same protein’s three-dimensional structure, suggesting that peptide 16661 is actually exposed.

As observed in *P. falciparum* antigens studies [34], *Mtb* H37Rv protein LpqG peptides’ relevant sequences in host-pathogen interaction were less recognised by patients than other sequences of same protein. The relevant sequence in our proposal (16661:^21^SGCDSHNSGSLGADPRQVTVY^40^) must therefore have changes (replacements) made to its structure to convert it into an immunogenic sequence capable of inducing a protective immune response against the original LpqG protein- derived sequence.

## 4. Materials and Methods

### 4.1. Bioinformatics Analysis of the LpqG Protein

In silico analysis of the LpqG protein sequence obtained from the TubercuList database (http://tuberculist.epfl.ch) led to defining protein characteristics and establishing a possible role in host-pathogen interaction. BLAST (basic local alignment search tool) software and MUSCLE multiple alignment tool [35] were initially used for determining LpqG protein homology amongst MTC strains due to the importance of studying proteins possibly involved in *Mtb* pathogenicity. ProtParam [36] was used for characterising the protein’s basic parameters and Gpos-PLoc [37], PA-SUB v.2.5. [38] and PSORTb v.2.0.4 [39] bioinformatics tools were used for predicting LpqG protein location, as our approach focuses on proteins on subcellular envelop (i.e., such tools have been validated for use with *Mtb* H37Rv proteins) [40].

Once it had been determined that the protein of interest was located on mycobacterial surface (by the presence of the LpqG signal peptide sequence), Signal P 4.0 [41], TatP 1.0 [42] and LipoP 1.0 [43] were used for analysing the secretion pathway by which it was exported to such location; SecretomeP 2.0 [44] was used for evaluating secretion by no-classical route. TMHMM 2.0 bioinformatics tool [45] was used for ascertaining the presence of transmembrane helices and NetOGlyc 4.0 [46] for determining whether LpqG had glycosylation sites.

### 4.2. lpqG gene Presence and Transcription

The presence and transcription of the *lpqG* gene encoding the Rv3623 protein was determined in *Mtb* H37Rv H37Rv (ATCC 27294), *Mtb* H37Ra (ATCC 25177) and *M. smegmatis* (ATCC 19420) grown on Middlebrook 7H9 medium (Difco Laboratories, Detroit, MI, USA) supplemented with oleic acid, albumin, dextrose and catalase (OADC), for 20 or 5 days and *M. bovis* (ATCC 19210) and *M. bovis* BCG (ATCC 27291) on pyruvic acid-enriched culture medium, without glycerol, incubated at 37 °C. Mycobacterial cells were harvested by spinning at 13,000 g for 40 min at 4 °C.

An Ultra Clean Microbial DNA isolation kit (MoBio Laboratories Inc., Carlsbad, CA, USA) was used for extracting bacterial genomic DNA (gDNA) from the aforementioned strains, following the manufacturer’s recommendations. Extracted DNA quality was verified by PCR using primers for the *hsp65* constitutive gene (forward 5’-ACCAACGATGGTGTGTCCAT-3’ and reverse 5’-CTTGTC GAACCGCATACCCT-3’). The following primers were used for corroborating *lpqG*gene presence and transcription: *rv3623* forward 5’-CAACGACATCGAGGTGAA-3’ and reverse 5’-GAGATCACC TTGCCTAGC-3’.

Sodium azide (10 mM) was added to the culture just before harvesting. The cell pellet was suspended in 2 mL cold lysis buffer per 200 mg wet weight of cells and sonicated twice for 15 min. Two volumes of Trizol (Invitrogen, Carlsbad, CA, USA) were added, followed by continuous extraction, according to the manufacturer’s recommendations. The final pellet was suspended in 100 µL sterile water and stored in aliquots at −80 °C; DNase I (Invitrogen, Carlsbad, CA, USA) was used for and treating RNA, following the manufacturer’s instructions. SuperScript III Reverse Transcriptase (Invitrogen, Carlsbad, CA, USA) was used for synthesising complementary DNA (cDNA). A negative synthesis control was included for each sample, substituting reverse transcriptase for diethyl pyrocarbonate (DEPC)-treated water. The previously described protocol required 1 µL cDNA as template for PCR amplification, using GoTaq DNA polymerase (Promega, Madison, WI, USA), 1.5 mM MgCl_2_, 1mM dNTPs, and 1 µL of each primer. Thermocycling conditions were 95 °C for 5 min, followed by 35 cycles at 56 °C for 30 s, 72 °C for 30 s and 95 °C for 30 s and a final extension step at 72 °C for 5 min. Amplifications were visualised on SYBR Safe stained 2% agarose gels (Invitrogen, Carlsbad, CA, USA).

### 4.3. LpqG Translation in Mycobacterium tuberculosis H37Rv

Once *lpqG*gene presence and transcription had been determined, the protein’s translation in *Mtb* H37Rv in normal culture conditions was verified; polyclonal serum was used for this. Briefly, female BALB/c mice were inoculated with 80 µg LpqG peptide 16665 (VAPQYSNPEPAGTATI TGYR) (BepiPred 1.0 Server identifying it as a B-cell epitope [46]), chemically coupled to BSA. Peptide 16660 (MIRLVRHSIALVAAGLAAALY) was also used; it has been identified as a T-cell epitope for this murine model by the NetMHCIIpan3.1 server [47]. Peptides-BSA were mixed with Freud’s incomplete adjuvant (Sigma, St. Louis, MO, USA) administered three times at 20-day intervals and pre-immune and post- third immunisation blood samples were taken.

LpqG presence and possible location in *Mtb*H37Rv protein was then determined by Western blot from the sera so obtained; the mycobacteria were sonicated and then wall, membrane and cytosol sub-cellular fractions were obtained using the lysis buffer (0.05 M potassium phosphate, 0.022% (*v*/*v*) β-mercaptoethanol, pH 6.5) [48]. The proteins were separated by 20% SDS-PAGE and transferred to a nitrocellulose membrane. The membrane was incubated with sera diluted 1:20 after washing and then incubated with anti-mouse IgG secondary antibody (Vector Laboratories, Burlingame, CA, USA) at 1:10,000 dilution, labelled with alkaline phosphatase and revealed with BCIP/NBT (Promega, Madison, WI, USA).

### 4.4. Identifying Synthetic Peptides Having High Specific LpqG Binding to Target Cells

Twelve 20-amino-acid-long, non-overlapping synthetic peptides covering the LpqG protein sequence were used in this study; they were obtained by solid phase multiple synthesis, purified by reverse phase high-resolution liquid chromatography (RP-HPLC) and characterised by MALDI-TOF mass spectrometry. A Tyr residue was added to peptides lacking it to enable Na^125^I radio-labelling. Receptor-ligand assays were then used for determining each peptide’s A549 alveolar epithelial and monocyte-derived U937 macrophage infection target cell binding capability, following previously described methodology [18,19,20,21]. Briefly, 1.2 × 10^6^ cell/well were incubated for 2 h with increasing concentrations of radio-labelled peptide (0–950 nM) and, after incubation, the cells were separated by spinning on a dioctyl phthalate/dibutyl phthalate (60:40) cushion. Cell-associated radioactivity was measured using a gamma counter (Cobra III, Packard Instrument Co., Meriden, CT, USA).

An assay involved adding an excess (40 µM) of the same non-radio-labelled peptide in the previously mentioned conditions was carried out simultaneously. Total binding was defined as the amount of radio-labelled peptides binding to cells in the absence of non-radio-labelled peptide whilst binding in the presence of non-radio-labelled peptide was called non-specific binding. The difference between total binding and inhibited binding represents specific binding; when the specific binding curve slope was greater than or equal to 1%, a peptide was considered to have high specific binding activity (i.e., a HABP).

To obtain physicochemical constants regarding HABP-cell interaction, saturation assays were carried out using a higher radio-labelled peptide range (0–8.000 nM), following a previously described protein peptide screening methodology. Thereby, were determined for each HABP Dissociation constants (K_D_), Hill coefficients (n_H_) and binding sites per cell.

### 4.5. Inhibiting Mycobacterial Entry to Infection Target Cells

The role of peptides identified as HABPs inhibiting *Mtb* H37Rv entry to infection target cells was evaluated. This involved 2.5 × 10^5^ cells/well incubated in RPMI supplemented with 10% heat-inactivated bovine foetal sera at 37 °C, 5% CO_2_ until a monolayer formed. The medium was then removed and the cells placed in contact with the peptides in increasing concentrations (2, 20 and 200 µM) for 2 h. Alveolar epithelial cells were incubated in aforementioned conditions; however, macrophages were placed at 4 °C to reduce their phagocytic activity [49].

The cells were then infected with *Mtb* H37Rv at 1:10 multiplicity of infection (MOI). Untreated cells were used as entry control and *Mtb* H37Rv lysate (200 μM) as inhibition control. Cells were left in contact with mycobacteria overnight at 37 °C in 5% CO_2_. The assays were done in triplicate. The medium was removed on the following day and the monolayer washed with PBS at pH 7.3; adhered cells and were dislodged using 0.3% trypsin, 0.017 mM EDTA and then neutralised with RPMI supplemented with 10% FBS. An aliquot was plated on Middlebrook 7H10 medium with OADC and incubated at 37 °C. A colony forming unit (CFU) assay was run 20 days later for calculating invasion rate by comparing infection control CFU estimated as 100% invasion. A second aliquot (400 µL) was fixed with cold 4% paraformaldehyde –0.05% glutaraldehyde overnight at 4 °C, washed with PBS and incubated in NH_4_Cl 50 mM for 2 h at 4 °C and then washed with PBS to be observed by fluorescence microscopy. Fluorescence microscopy analysis was possible since *Mtb* H37Rv expressing GFP was used in this assay (i.e., for visualising mycobacterial entry to target cells).

An MTT cell viability assay was used for evaluating LpqG peptides’ cytotoxic effect; it involved placing 5 × 10^4^ cells/well for 2 h with 20 and 200 µM each peptide in PBS, using the afore-mentioned incubation conditions. Triton X-100 was used as cytotoxicity control; 10 µL MTT were then added for 12 h. The resulting formazan crystals were dissolved in SDS and absorbance read at 570 nm.

### 4.6. Evaluating LpqG Peptide Antigenicity

The 12 LpqG peptides’ antigenic capability was evaluated by ELISA using 5 asymptomatic patients’ sera samples (having had positive Quantiferon tests), classified as latent tuberculosis (LTB), 5 sera samples from patients having positive conventional microbiological diagnostic test and positive *Mtb* culture, classified as active tuberculosis (ATB), and 5 healthy controls (HC), having had a negative Quantiferon test and no background of previous contact with *Mtb*. The Quantiferon test used for classifying the patients determined interferon-γ concentration in plasma by ELISA as response to saline solution (negative control), PHA (mitogen control) and MTC strain ESAT-6 and CFP-10 and TB7.7 antigens; making this a highly specific test compared to conventional tuberculin test [50]. Blood samples were collected by venipuncture and all donors gave their written consent before donating blood. The study protocol was approved by the School of Medicine and Health Sciences of Universidad del Rosario (Bogotá, Colombia) Ethics’ Committee (minutes 197, 13 October 2011).

The ELISA assay involved LpqG peptides (10 µg/mL) being immobilised in triplicate in 96-well polystyrene microtitre plates, incubating for 12 h at 4 °C. Non-specific sites were then blocked with 5% skimmed milk in PBS-Tween for 2 h and washed with PBS-0.5% Tween and water. Patients’ plasma was placed after at 1:100 dilution for 2 h at 37 °C. After washing, anti-human IgG secondary antibody was added at 1:5000 dilution, incubated at 37 °C for 1 h and revealed using the TBM-peroxidase system for reading at 450 nm. *Mtb* H37Rv culture lysate and supernatant were used as controls.

### 4.7. Determining LpqG Peptide Secondary Structure

As a protein’s function is closely linked to its structure, complete protein structure was predicted and LpqG protein peptides’ secondary structure experimentally determined. PSIPRED [51] was used for predicting secondary structure from the reported LpqG protein sequence and Phyre^2^ [27] for modelling tertiary structure; this was validated by Swiss model workspace assessment [52]; this was followed by using Amber software [53] for analysing energy minimisation. Circular dichroism (CD) was used for experimental determination with 5 × 10^−6^ M peptide solution in 30% trifluoroethanol (*v*/*v*), TFE (used as structure stabiliser). Samples were read in triplicate on a J-810 spectropolarimeter (JASCO, Easton, MD, USA) giving ellipticity values (θ) measured in millidegrees (mdeg) on a 190–260 nm spectral sweep. SELCON3, CDSSTR and CONTINLL [31] were used for data deconvolution, estimating each peptide’s secondary structure elements expressed as α-helices, folded β-sheets and random coils.

## 5. Conclusions

Tuberculosis continues gaining importance amongst all diseases because it has a negative impact on public health, in addition to the BCG vaccine’s current ineffectiveness causing increased *Mtb*–related morbidity-mortality. Our approach involves using such in-house proven methodology for identifying sequences of proteins selected from *Mtb* H37Rv that could lead to developing a fully-protective, multi-epitope anti-TB vaccine. Here, regarding the LpqG protein was identified the sequence ^21^SGCDSHNSGSLGADPRQVTVY^40^ which can bind specifically and with high affinity to target cells and are able to inhibit bacilli entry (in in vitro assays) as an attractive basic components for such efficient anti-TB vaccine. Taking into account that HABPs are poorly immunogenic, next step would be to modify their sequences following the reported methodology, which has been thoroughly used for malaria in order to render them immunogenic and protection inducing [34].

## Figures and Tables

**Table 1:** LpqG (Rv3623) protein features related to subcellular localisation prediction and major characteristics.

Protein		Rv3623
TubercuList description		probable conserved lipoprotein LPQG
Molecular weight		24,836.80 Da
Theoretical pI		5.52
Instability index		21.64 stable
Aliphatic index		93.54
Grand average of hydropathicity (GRAVY)		−0.055
Subcellular localisation	PA-SUB v.2.5.	Not cytoplasm
		Not extracellular
		Not plasma membrane
	Gpos-Ploc	Plasma membrane
	PSORTb v.2.0.4	Unknown
LipoP 1.0	Score	25.1723
	Cleavage site	22–23 Pos + 2 = D
Phobius	TM/SP/prediction	0/Y/n9-20c25/26o
TMHMM 2.0	ExpAA	0.24
	First60	0.24
	PredHel	0
	Topology	outer
SignalP 4.0	Signal peptide probability	0.939
	Max. cleavage site probability	0.697
	Cleavage site	25–26
NetOGlyc 4.0		Thr: 96, 97, 100, 207 Ser: 218

PredHel: amount of transmembrane helices predicted by TMHMM 2.0. Regarding Phobius predictions: TM indicates the amount of predicted transmembrane segments, SP: indicates if there is a predicted signal peptide.

**Table 2:** Physicochemical constants in LpqG protein peptide HABP-cell interaction.

	U937	A549		
HABP	16661	16663	16664	16665
Dissociation constant (K_D_), nM	2700	2800	2000	2000
Hill coefficient (n_H_)	1.5	1.4	3.0	1.4
Binding sites per cell	5 × 10^6^	9 × 10^6^	7 × 10^6^	3 × 10^6^

## References

[1] Ernst J.D. (2012). The immunological life cycle of tuberculosis. Nat. Rev. Immunol..

[2] World Health Organization (2017). WHO Global Tuberculosis Report 2017.

[3] Mangtani P., Abubakar I., Ariti C., Beynon R., Pimpin L., Fine P.E., Rodrigues LC, Smith P.G., Lipman M., Whiting P.F. (2014). Protection by BCG vaccine against tuberculosis: A systematic review of randomized controlled trials. Clin. Infect. Dis..

[4] Palomino J.C., Martin A. (2014). Drug Resistance Mechanisms in *Mycobacterium tuberculosis*. Antibiotics.

[5] Dockrell H.M., Smith S.G. (2017). What Have We Learnt about BCG Vaccination in the Last 20 Years?. Front. Immunol..

[6] Boggiano C., Eichelberg K., Ramachandra L., Shea J., Ramakrishnan L., Behar S., Ernst J.D., Porcelli S.A., Maeurer M., Kornfeld H. (2017). “The Impact of *Mycobacterium tuberculosis* Immune Evasion on Protective Immunity: Implications for TB Vaccine Design”—Meeting report. Vaccine.

[7] Awuh J.A., Flo T.H. (2017). Molecular basis of mycobacterial survival in macrophages. Cell. Mol. Life Sci..

[8] Behar S.M., Divangahi M., Remold H.G. (2010). Evasion of innate immunity by *Mycobacterium tuberculosis*: is death an exit strategy?. Nat. Rev. Microbiol..

[9] Dorhoi A., Reece S.T., Kaufmann S.H.E. (2011). For better or for worse: The immune response against *Mycobacterium tuberculosis* balances pathology and protection. Immunol. Rev..

[10] Sani M., Houben E.N., Geurtsen J., Pierson J., de Punder K., van Zon M., Wever B., Piersma S.R., Jimenez C.R., Daffe M. (2010). Direct visualization by cryo-EM of the mycobacterial capsular layer: a labile structure containing ESX-1-secreted proteins. PLoS Pathog..

[11] Gu S., Chen J., Dobos K.M., Bradbury E.M., Belisle J.T., Chen X. (2003). Comprehensive proteomic profiling of the membrane constituents of a *Mycobacterium tuberculosis* strain. Mol. Cell. Proteom. MCP.

[12] Cole S.T., Brosch R., Parkhill J., Garnier T., Churcher C., Harris D., Gordon S.V., Eiglmeier K., Gas S., Barry C.E. (1998). Deciphering the biology of *Mycobacterium tuberculosis* from the complete genome sequence. Nature.

[13] Sinha S., Arora S., Kosalai K., Namane A., Pym A.S., Cole S.T. (2002). Proteome analysis of the plasma membrane of *Mycobacterium tuberculosis*. Comp. Funct. Genom..

[14] Rezwan M., Grau T., Tschumi A., Sander P. (2007). Lipoprotein synthesis in mycobacteria. Microbiology.

[15] Sutcliffe I.C., Harrington D.J. (2004). Lipoproteins of *Mycobacterium tuberculosis*: An abundant and functionally diverse class of cell envelope components. FEMS Microbiol. Rev..

[16] Malen H., Pathak S., Søfteland T., de Souza G.A., Wiker H.G. (2010). Definition of novel cell envelope associated proteins in Triton X-114 extracts of *Mycobacterium tuberculosis* H37Rv. BMC Microbiol..

[17] Malen H., Berven F.S., Softeland T., Arntzen M.O., D’Santos C.S., De Souza G.A., Wiker H.G. (2008). Membrane and membrane-associated proteins in Triton X-114 extracts of Mycobacterium bovis BCG identified using a combination of gel-based and gel-free fractionation strategies. Proteomics.

[18] Ocampo M., Patarroyo M.A., Vanegas M., Alba M.P., Patarroyo M.E. (2013). Functional, biochemical and 3D studies of *Mycobacterium tuberculosis* protein peptides for an effective anti-tuberculosis vaccine. Crit. Rev. Microbiol..

[19] Díaz D.P., Ocampo M., Pabón L., Herrera C., Patarroyo M.A., Muñoz M., Patarroyo M.E. (2016). *Mycobacterium tuberculosis* PE9 protein has high activity binding peptides which inhibit target cell invasion. Int. J. Biol. Macromol..

[20] Díaz D.P., Ocampo M., Varela Y., Curtidor H., Patarroyo M.A., Patarroyo M.E. (2017). Identifying and characterising PPE7 (Rv0354c) high activity binding peptides and their role in inhibiting cell invasion. Mol. Cell. Biochem..

[21] Rodriguez D.C., Ocampo M., Reyes C., Arevalo-Pinzon G., Munoz M., Patarroyo M.A., Patarroyo M.E. (2016). Cell-Peptide Specific Interaction Can Inhibit *Mycobacterium tuberculosis* H37Rv Infection. J. Cell. Biochem..

[22] Li W., Cowley A., Uludag M., Gur T., McWilliam H., Squizzato S., Park Y.M., Buso N., Lopez R. (2015). The EMBL-EBI bioinformatics web and programmatic tools framework. Nucleic Acids Res..

[23] Gasteiger E., Hoogland C., Gattiker A., Duvaud S., Wilkins M.R., Appel R.D., Bairoch A. (2005). Protein Identification and Analysis Tools on the ExPASy Server. The Proteomics Protocols Handbook.

[24] Kyte J., Doolittle F. (1982). A simple method for displaying the hydropathic character of a protein. J. Mol. Biol..

[25] Shen H.B., Chou K.C. (2009). Gpos-mPLoc: A top-down approach to improve the quality of predicting subcellular localization of Gram-positive bacterial proteins. Protein Pept. Lett..

[26] Käll L., Krogh A., Sonnhammer E.L. (2007). Advantages of combined transmembrane topology and signal peptide prediction—The Phobius web server. Nucleic Acids Res..

[27] Kelley L.A., Mezulis S., Yates C.M., Wass M.N., Sternberg M.J. (2015). The Phyre^2^ web portal for protein modeling, prediction and analysis. Nat. Protoc..

[28] Benkert P., Biasini M., Schwede T. (2011). Toward the estimation of the absolute quality of individual protein structure models. Bioinformatics.

[29] Arnold K., Bordoli L., Kopp J., Schwede T. (2006). The SWISS-MODEL Workspace: A web-based environment for protein structure homology modelling. Bioinformatics.

[30] Kelly S.M., Jess T.J., Price N.C. (2005). How to study proteins by circular dichroism. BBA Proteins Proteom..

[31] Sreerama N., Woody R.W. (2000). Estimation of protein secondary structure from circular dichroism spectra: Comparison of CONTIN, SELCON, and CDSSTR methods with an expanded reference set. Analy. Biochem..

[32] Babu M.M., Priya M.L., Selvan A.T., Madera M., Gough J., Aravind L., Sankaran K. (2006). A database of bacterial lipoproteins (DOLOP) with functional assignments to predicted lipoproteins. J. Bacteriol..

[33] Steentoft C., Vakhrushev S.Y., Joshi H.J., Kong Y., Vester-Christensen M.B., Schjoldager K.T., Lavrsen K., Dabelsteen S., Pedersen N.B., Marcos-Silva L. (2013). Precision mapping of the human *O*-GalNAc glycoproteome through SimpleCell technology. EMBO J..

[34] Patarroyo M.E., Bermudez A., Patarroyo M.A. (2011). Structural and immunological principles leading to chemically synthesized, multiantigenic, multistage, minimal subunit-based vaccine development. Chem. Rev..

[35] Edgar R.C. (2004). MUSCLE: A multiple sequence alignment method with reduced time and space complexity. BMC Bioinform..

[36] Bairoch A., Apweiler R., Wu C.H., Barker W.C., Boeckmann B., Ferro S., Gasteiger E., Huang H., Lopez R., Magrane M. (2005). The Universal Protein Resource (UniProt). Nucleic Acids Res..

[37] Chou K.C., Shen H.B. (2008). Cell-PLoc: A package of Web servers for predicting subcellular localization of proteins in various organisms. Nat. Protoc..

[38] Lu Z., Szafron D., Greiner R., Lu P., Wishart D.S., Poulin B., Anvik J., Macdonell C., Eisner R. (2004). Predicting subcellular localization of proteins using machine-learned classifiers. Bioinformatics.

[39] Gardy J.L., Laird M.R., Chen F., Rey S., Walsh C.J., Ester M., Brinkman F.S. (2005). PSORTb v.2.0: Expanded prediction of bacterial protein subcellular localization and insights gained from comparative proteome analysis. Bioinformatics.

[40] Restrepo-Montoya D., Vizcaíno C., Niño L.F., Ocampo M., Patarroyo M.E., Patarroyo M.A. (2009). Validating subcellular localization prediction tools with mycobacterial proteins. BMC Bioinform..

[41] Petersen T.N., Brunak S., von Heijne G., Nielsen H. (2011). SignalP 4.0: Discriminating signal peptides from transmembrane regions. Nat. Methods.

[42] Bendtsen J.D., Nielsen H., Widdick D., Palmer T., Brunak S. (2005). Prediction of twin-arginine signal peptides. BMC Bioinform..

[43] Juncker A.S., Willenbrock H., Von Heijne G., Brunak S., Nielsen H., Krogh A. (2003). Prediction of lipoprotein signal peptides in Gram-negative bacteria. Protein Sci..

[44] Bendtsen J.D., Kiemer L., Fausboll A., Brunak S. (2005). Non-classical protein secretion in bacteria. BMC Microbiol..

[45] Sonnhammer E.L., von Heijne G., Krogh A. (1998). A hidden Markov model for predicting transmembrane helices in protein sequences. Proc. ISMB-98 Proc..

[46] Larsen J.E., Lund O., Nielsen M. (2006). Improved method for predicting linear B-cell epitopes. Immunome Res..

[47] Andreatta M., Karosiene E., Rasmussen M., Stryhn A., Buus S., Nielsen M. (2015). Accurate pan-specific prediction of peptide-MHC class II binding affinity with improved binding core identification. Immunogenetics.

[48] Rezwan M., Laneelle M.A., Sander P., Daffe M. (2007). Breaking down the wall: Fractionation of mycobacteria. J. Microbiol. Methods.

[49] Bermudez L.E., Goodman J. (1996). *Mycobacterium tuberculosis* invades and replicates within type II alveolar cells. Infect. Immun..

[50] Cascante J., Pascal I., Eguía V., Hueto J. (2007). Diagnosis of tuberculosis infection. SciELO.

[51] Jones D.T. (1999). Protein secondary structure prediction based on position-specific scoring matrices. J. Mol. Biol..

[52] Biasini M., Bienert S., Waterhouse A., Arnold K., Studer G., Schmidt T., Kiefer F., Gallo Cassarino T., Bertoni M., Bordoli L. (2014). SWISS-MODEL: Modelling protein tertiary and quaternary structure using evolutionary information. Nucleic Acids Res..

[53] Salomon-Ferrer R., Gotz A.W., Poole D., Le Grand S., Walker R.C. (2013). Routine Microsecond Molecular Dynamics Simulations with AMBER on GPUs. 2. Explicit Solvent Particle Mesh Ewald. J. Chem. Theory Comput..

